# Six new species of *Agrilus* Curtis, 1825 (Coleoptera, Buprestidae, Agrilinae) from the Oriental Region related to the emerald ash borer, *A. planipennis* Fairmaire, 1888 and synonymy of *Sarawakita* Obenberger, 1924

**DOI:** 10.3897/zookeys.239.3966

**Published:** 2012-11-08

**Authors:** Eduard Jendek, Maria Lourdes Chamorro

**Affiliations:** 1 Ottawa Plant Laboratory, Canadian Food Inspection Agency, K.W. Neatby Bldg., 960 Carling Avenue, Ottawa, Ontario, K1A 0C6, Canada; 2 Systematic Entomology Laboratory, United States Department of Agriculture, National Museum of Natural History, 10th & Constitution Ave NW, Washington, DC 20560, USA

**Keywords:** *Agrilus*, Buprestidae, emerald ash borer, new species, synonym, taxonomy, nomenclature, Asia

## Abstract

Six new species of *Agrilus* Curtis, 1825 with affinities to the emerald ash borer, *Agrilus planipennis* Fairmaire, 1888, are described from the Oriental Region: *Agrilus crepuscularis* **sp**. **n**. (Malaysia); *Agrilus pseudolubopetri* **sp**.** n**. (Laos); *Agrilus sapphirinus* **sp**. **n**.(Laos); *Agrilus seramensis* **sp**. **n**.(Indonesia); *Agrilus spineus* **sp**. **n**. (Malaysia); and *Agrilus tomentipennis* **sp**. **n**. (Laos). The genus *Sarawakita* Obenberger, 1924 **syn**. **nov**. is considered a junior synonym of *Agrilus*.

## Introduction

The current study stems from an international, multi-agency effort between the following institutions (listed in alphabetical order): the Canadian Food Inspection Agency, the Chinese Academy of Sciences Institute of Zoology, the United States Department of Agriculture, and the Zoological Institute, Russian Academy of Sciences to understand the evolutionary relationships and biology of the highly invasive *Agrilus planipennis* Fairmaire, 1888 (emerald ash borer – EAB) and its relatives. This effort aims to determine, define, and illustrate the characters that enable identification of EAB and a core group of related species; make predictions about potential new invasive species with similar evolutionary histories and adaptations; educate the public and other scientists; and contribute knowledge needed to develop control strategies to manage outbreaks. A comprehensive, illustrated identification manual presenting these findings is underway. In this paper we describe six new species related to *Agrilus planipennis* and propose new taxonomic and nomenclatural acts discovered during the course of our study.

## Materials and methods

Terminology, morphology, format and style of descriptions follow Jendek and Grebennikov (2011). Square brackets “[ ]” are used for our remarks and addenda. The following equipment was used for observation and imaging: Leica (Wetzlar, Germany) MZ Apo stereomicroscope and Zeiss (Oberkochen, Germany) Discovery v20 stereomicroscope with AxioCam HRc, respectively.

Abbreviations for collections

EJCBJendek, E., Bratislava, Slovak Republic [presently in Ottawa, Canada]


MNHNMuséum National d’Histoire Naturelle, Paris, France (Bruneau de Miré, I; Mantilleri, A.)


NMPCNárodní Museum (Natural History), Prague, Czech Republic (Kubáň, V.)


USNMNational Museum of Natural History, Washington D.C., USA (Lingafelter, S.W.)


## Taxonomic section

*Agrilus* Curtis, 1825

= *Sarawakita* Obenberger, 1924 **syn. n.**

Obenberger 1924: 39–40, figs 15, 41 (proposed as genus; Type species: *Sarawakita latifrons* Obenberger, 1924 fixed by original designation and monotypy) – Obenberger 1936: 1085 (world catalog) – Kubáň et al. 2000: 196 (valid genus; *Agrilini*) – Bellamy 2003: 2380 (valid genus; Agrilini *incertae sedis*)

### Agrilus hewitti

Kerremans, 1912 comb. rest.

http://species-id.net/wiki/Agrilus_hewitti

Figs 1–8

Agrilus hewitti Kerremans, 1912: 74 (*Agrilus*, description) – Obenberger 1936: 1085 (world catalog) – Obenberger 1960: 125–126 (type examination; redescription) – Jendek 2006: 34 (*Sarawakita*; lectotype designation; synonymy) – Bellamy 2008: 2380 (*Sarawakita*; world catalog).
Agrilus latifrons Obenberger, 1924: 40 (*Sarawakita*, description) – Jendek 2006: 34 (*Sarawakita*; synonym of *hewitti*; lectotype designation) – Bellamy 2008: 2380 (*Sarawakita*; synonym of *hewitti*).

#### Material examined.

Type material. See Jendek (2006).

**Other material.** 1 (EJCB): “Sarawak 1897”; 1 (EJCB): “Malaysia, Pahang, 2000, Cameron Highlands, Tanah Rata, 1600m, J. Horák leg. 26.1.–10.2.”

**Figures 1–8.**
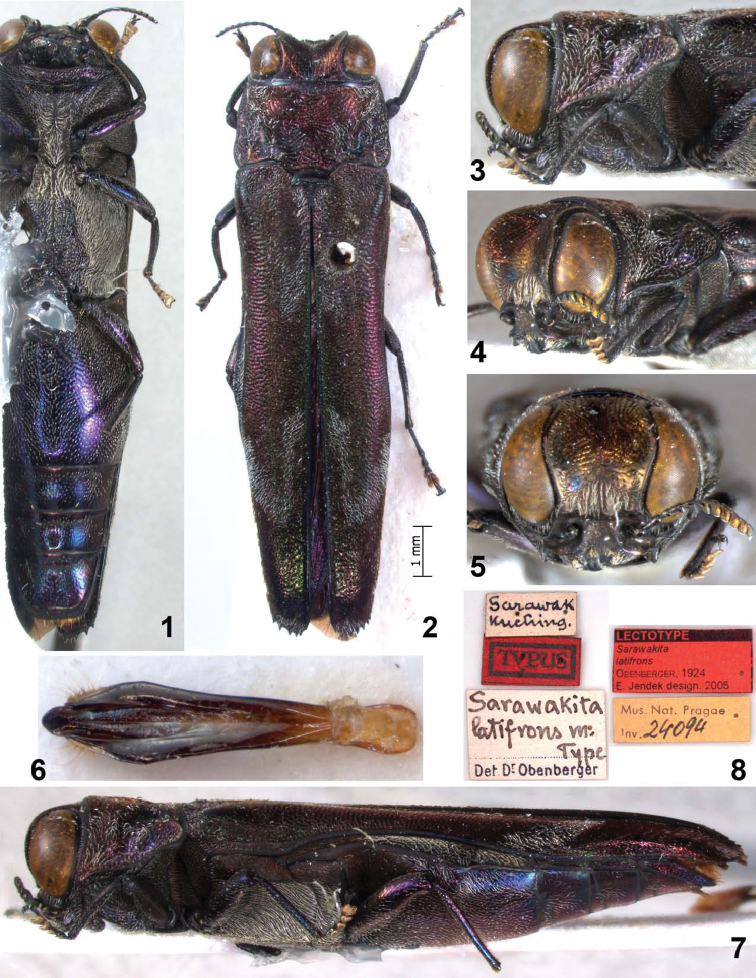
*Agrilus hewitti* Kerremans. Holotype: **1** ventral view **2** dorsal view **3** lateral view of head and pronotum **4** oblique-lateral view of head and pronotum **5** anterior view of head **6** dorsal view of aedeagus **7** lateral view **8** type labels.

### Agrilus daillieri

Baudon, 1965

http://species-id.net/wiki/Agrilus_daillieri

Figs 9–11

Agrilus daillieri Baudon, 1965: 223-224 (*Agrilus*, description) – Baudon 1963: 54 ([Note: Unavailable name, cited without characters]) – Descarpentries and Villiers 1967: 149 (*sinensis* species group) – Baudon 1968: 135, 168 (characters in key; Laos) – Ohmomo 2002: 23 (faunal records; Thailand) – Bellamy 2008: 2057 (world catalog).

#### Material examined.

Type material. Holotype ♂, (MHNB): “Pak Ca Dinh 15.v.[19]63 [h] Laos (Baudon) [p] \ Type [p] [red label] \ Agrilus daillieri mihi Type [h] A. Baudon det. [p] [blue label]”.

**Other material.** 1 (ZIN): “Vietnam, Vinh-Phu Prov. Tam-Dao V–VI.1997, N. L. Orlov leg.”; 1 (EJCB): “N Vietnam (Tonkin) pr. Hoang Lien Son, SA PA 11–15.v.1990, Vit Kubáň leg.”; 1 (EJCB): “Vietnam, Tam Dao, Vinh-Phu Pr., 3–11.6.1985, Navrátil lgt., Collectio Vit Kubáň”; 1 (EJCB): “Vietnam, 1100–1700, 22.18N, 103.50E, W SaPa, 29.V–11.VI.1996, lg. K.W. Anton”; 1 (EJCB): “N. Vietnam, 21°27N, 105°39E, 70 km NW of Hanoi, Tam Dao, 9–19.v.1996, 900–1200m, Dembicky & Pacholátko leg.”; 1 (EJCB): “North Vietnam, Tam Dao, 28.vii.1997”.

#### Remarks.

Upon examination of the material mentioned above and the type specimens of *Agrilus hewitti*, *Sarawakita latifrons* and *Agrilus daillieri* we have come to the conclusion that *Sarawakita* should be treated as a junior synonym of *Agrilus*.

*Agrilus daillieri* and *Agrilus hewitti* are very closely related, large (> 10 mm) and robust species which share many morphological features with *Agrilus planipennis*. Their taxonomic position will be analyzed in detail in the upcoming revision.

**Figures 9–11.**
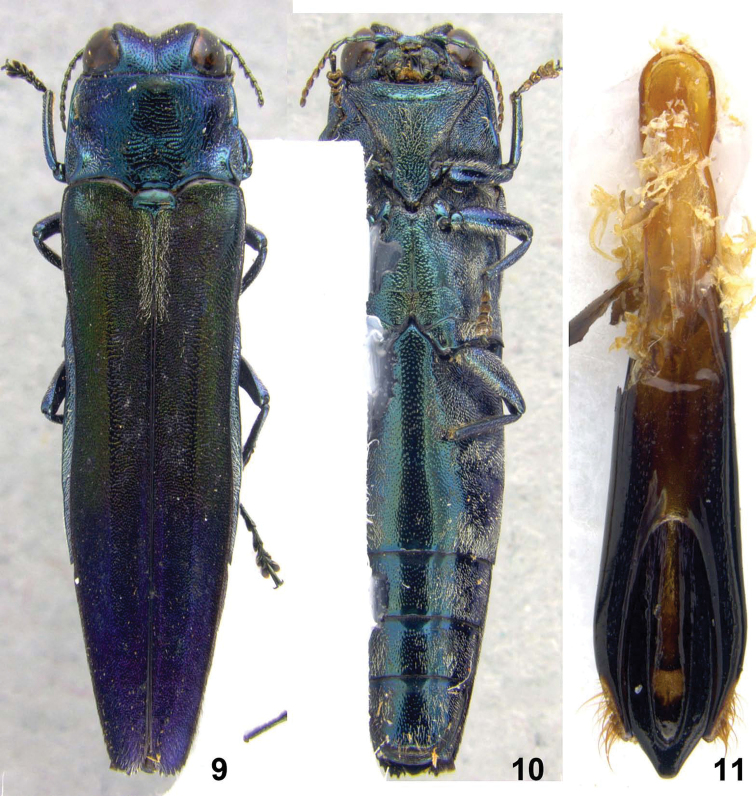
*Agrilus daillieri*Baudon. Holotype: **9** dorsal view **10** ventral view **11** dorsal view aedeagus.

### Agrilus crepuscularis

Jendek & Chamorro sp. n.

urn:lsid:zoobank.org:act:E04AB2A3-B27B-4B31-A6A4-123E124B7292

http://species-id.net/wiki/Agrilus_crepuscularis

Figs 12 23

#### Diagnosis.

This species resembles *Agrilus planipennis* by the body shape; transverse and trapezoid pronotum; obvious medial pronotal impression; very narrow marginal and submarginal interspace; rounded elytral apices; and by the small scutellum. *Agrilus crepuscularis* sp. n. can be distinguished from *Agrilus planipennis* mainly by the missing prehumerus; presence of obvious, yellow pubescence ventrally and by the rounded not spined apex of pygidium.

#### Description.

BODY: Size: 10 mm (Holotype); Shape: cuneiform; Build: slender.

HEAD: Shape: obviously flat; Medial impression (depth): deep; Medial impression (extent): vertex; **Epistoma**: with raised upper margin; **Frons**: Shape: markedly convex; Outline: protruding from head outline; **Vertex**: Outline: slightly protruding from head outline; **Sculpture**: punctures, semispherical, dense, rough; **Eyes**: Size: large; Shape: protruding from head outline; Lower margin: in line with antennal socket; **Antennae**:Length: moderate; Shape: slender.

PRONOTUM: Shape: visually square; Sides: markedly arcuate; Maximal width: at middle; **Anterior** **margin**: narrower than posterior; **Anterior lobe**: moderate; Shape: arcuate; Position: at level with anterior pronotal angles; **Posterior angles**: Apex: blunt, Shape: obtuse; **Disk**: Convexity: flat, **Disk impressions**: Presence: medial and lateral, Medial impressions (shape): entire, Medial impressions (depth): deep; Lateral impressions (Depth and width): shallow and broad; **Prehumerus**: absent; **Marginal and submarginal carinae**: Interspace: narrow; Convergence: strongly convergent; Junction: present; **Scutellum**: Size: small, Disk: impressed, Scutellar carina: present.

ELYTRA: Color: unicolored; **Humeral** **carina** absent; **Apices**: Arrangement: separate; Width: wide. Shape: arcuate.

STERNUM: **Prosternum**: with long yellow pubescence in males; **Prosternal lobe**: Size: moderately sized; Anterior margin: arcuately emarginate; Emargination wide and moderately deep; **Prosternal process**: Shape: dilated; Sides: arcuate; Angles: acute; Disk flat; **Metasternal projection**: flat.

ABDOMEN: **Sternal groove**: Shape on apex of last ventrite: arcuate; **Pygidium**:Apicalmargin: arcuate.

LEGS: **Metatarsus**: somewhat longer than mesotarsus; **Tarsomere** **1**: subequal to or longer than 2–4 combined.

GENITALIA: **Aedeagus**:Symmetry: symmetrical.

#### Type locality.

Malaysia, Pahang state, 35 km Southwest Kuala Rompin, 2.617N, 103.337E, Endau Rompin State Park.

#### Type specimens.

**Holotype**, ♂, (EJCB): “Malaysia, Pahang, 28.ii–13.iii, 35 km SW Kuala Rompin, 2.617N, 103.337E, 50 m, Endau Rompin State Park, E. Jendek leg. 2011”.

#### Distribution.

Malaysia: Pahang state.

#### Etymology.

The specific name is derived from the Latin *crepusculum* (twilight). It refers to the collecting circumstances with the holotype landing on the sheet when collecting at light.

**Figures 12–18.**
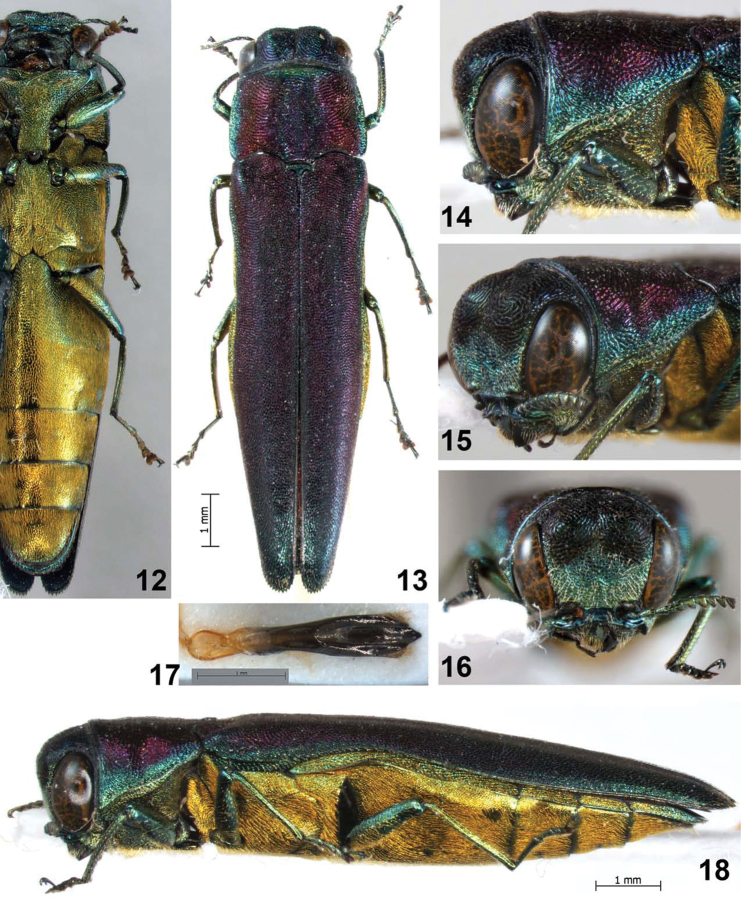
*Agrilus crepuscularis* Jendek & Chamorro, sp. n. Holotype male: **12** ventral view **13** dorsal view **14** lateral view of head and pronotum **15** oblique-lateral view of head and pronotum **16** anterior view of head **17** dorsal view of aedeagus **18** lateral view.

**Figures 19–23.**
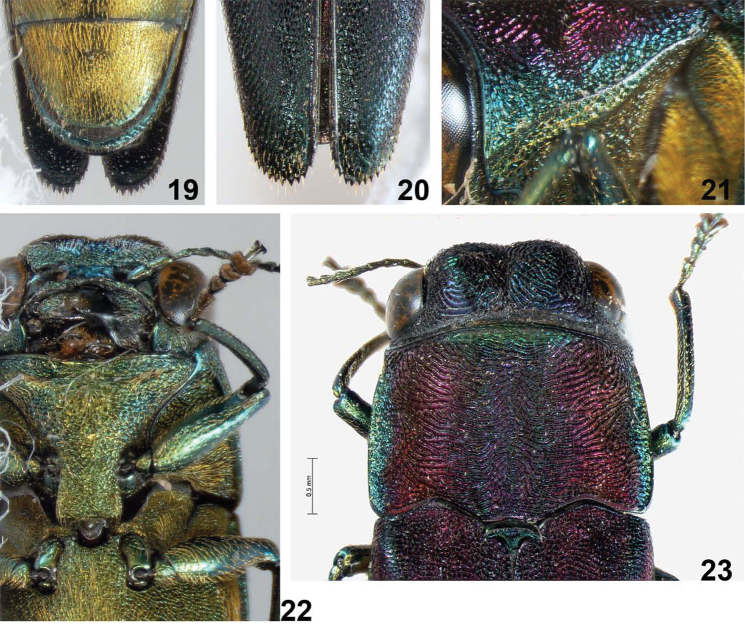
*Agrilus crepuscularis* Jendek & Chamorro, sp. n. Holotype male: **19** last abdominal ventrite **20** elytral apices **21** oblique-lateral view of marginal and submarginal carinae **22** ventral view of head and prosternum **23** dorsal view of head and pronotum.

### Agrilus pseudolubopetri

Jendek & Chamorro sp. n.

urn:lsid:zoobank.org:act:7E1BC733-2C05-4D96-94FA-0964E91548E2

http://species-id.net/wiki/Agrilus_pseudolubopetri

**Figures 24–25.**
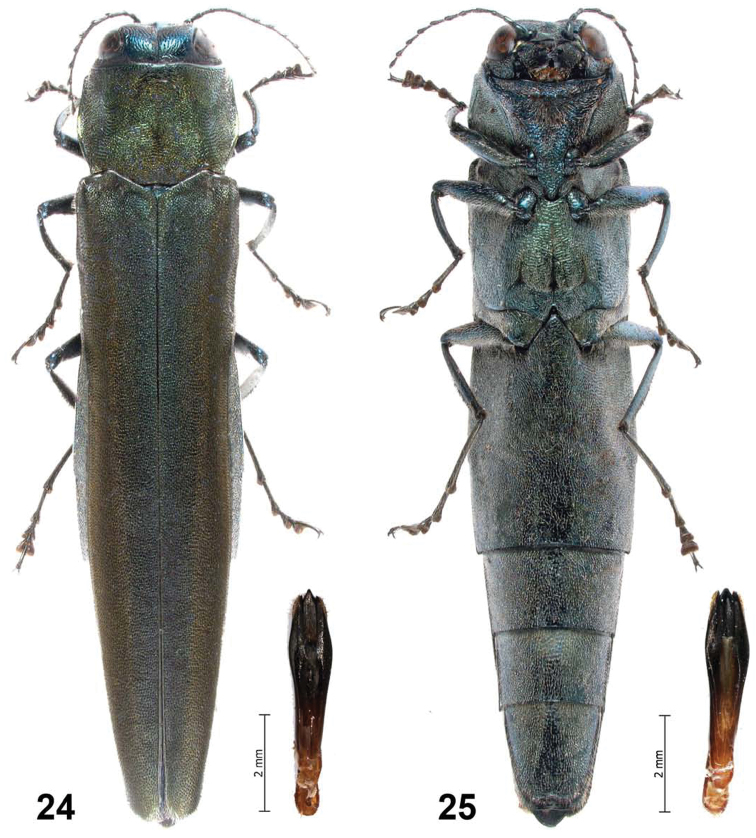
*Agrilus pseudolubopetri* Jendek & Chamorro, sp. n. Male habitus and aedeagus: **24** dorsal view **25** ventral view.

Figs 24 [Fig F6] [Fig F7] 39

#### Diagnosis.

The male resembles *Agrilus lubopetri* Jendek, 2000 in color, shape and size; however, the following characters distinguish the males of the two species: *Agrilus pseudolubopetri* sp. n. does not have expanded elytral apices and lacks white pubescence; the interspace between marginal and submarginal pronotal carinae is broader anteriorly in *Agrilus pseudolubopetri*; and the aedeagus is broader subapically. Female can be distinguished from females of *Agrilus lubopetri* by larger, more robust size, purple color (sometimes green to copper), and by unexpanded elytral apices. The orange pubescence on the pronotal sides of *Agrilus pseudolubopetri* is markedly less extensive than that in *Agrilus lubopetri*.

#### Description.

BODY: Size: 14–18 mm (Holotype 17 mm); Shape: cuneiform; Build: slender.

HEAD: Shape: obviously flat; **Medial impression**: deep; Extent: frons; **Epistoma**: with raised upper margin; **Frons**: Shape: flat; Outline: not protruding from head outline; **Vertex**:Outline: not protruding from head outline; **Sculpture**:punctures; Density: sparse; Intensity: superficial; **Eyes**: Size: large; Shape: not protruding from head outline; lower margin below antennal socket; **Antennae**: **Length**: long (males), medium (females), Shape: slender.

PRONOTUM: Shape: transverse; Sides: markedly arcuate; Maximal width: at middle; Anterior margin: wider than posterior; **Anterior lobe**: vague; Shape: arcuate; Position: at level with anterior pronotal angles; **Posterior angles**: Apex: sharp, Shape: acute; **Disk**: Convexity: flat; **Disk impressions**: Presence: medial and lateral, medial impression (shape): entire; lateral impressions: shallow and wide; **Prehumerus**: absent; **Marginal and submarginal carinae**: Interspace: narrow; Convergence: strongly convergent; Junction: present; **Scutellum**: Size: rudimentary, Disk: not impressed, Scutellar carina: obsolete or present.

ELYTRA: Color: unicolored; Humeral carina absent; **Apices**: Arrangement: separate; Shape: subangulate, Modifications: margin denticulate; Elytral pubescence: distal only; Distal: apical. STERNUM: Sexual modification in male: with longer white pubescence; **Prosternal lobe**:Size: moderate; Anterior margin: arcuately emarginate; Emargination: Depth: moderately deep; Width: wide; **Prosternal process**: Size: moderate; Shape: narrowed; Angles: absent; Disk: flat; **Metasternal** **projection**: flat.

ABDOMEN: **Sternal groove**:Shape on apex of last ventrite: arcuate, Depth: shallow; Width: narrow; Pygidium (apical margin): arcuate; **Last ventrite** (apical margin): subtruncate.

LEGS: **Metatarsus**: distinctly longer than mesotarsus; **Metatarsomere** 1: longer than 2–4 combined.

GENITALIA: **Aedeagus**: Symmetry: symmetrical; Ovipositor: elongate.

#### Type locality.

Northeastern Laos, Hua Phan Province, ~20°12'N, 104°01'E, Phu Phan Mt.

#### Type specimens.

**Holotype**, ♂, (EJCB): “LAOS-NE, Hua Phan prov. ~20°12'N, 104°01'E, PHU PHAN Mt., 1500–1900m, 17 v.–3.vi.2007, Vit. Kubáň leg.”. **Paratypes**: 2 ♂, 1 ♀, (EJCB): “LAOS-NE, Houa Phan prov., 20°12'N, 104°01'E, 20°13.5'N, 103°59'.5E, Ban Saluei → Phou Pane Mt., 1340–1870m, 15.iv.–15.v.2008, Lao collectors leg.”; 50 ♂, 45 ♀ (USNM); 25 ♂, 38 ♀ (ECJB): “LAOS-NE, Hua Phan Province, Ban Saleui, Phou Pan (Mt), 20°12'N, 104°01'E, 7.iv–25.v.2010, 1300–1900m, leg. C. Holzschuh”. 42 ♂, 53 ♀ (USNM): “LAOS-NE, Hua Phan Province, Ban Saleui, Phou Pan (Mt), 20°12'N, 104°01'E, 1–31.v.2011, 1300–1900m, leg. C. Holzschuh”.

#### Distribution.

Laos: Hua Phan Province.

#### Etymology.

The specific epithet is a combination of the Greek adjective *pseudos* (false, lie) and –*lubopetri*. Name indicates relation to *Agrilus lubopetri*.

**Figures 26–27. F6:**
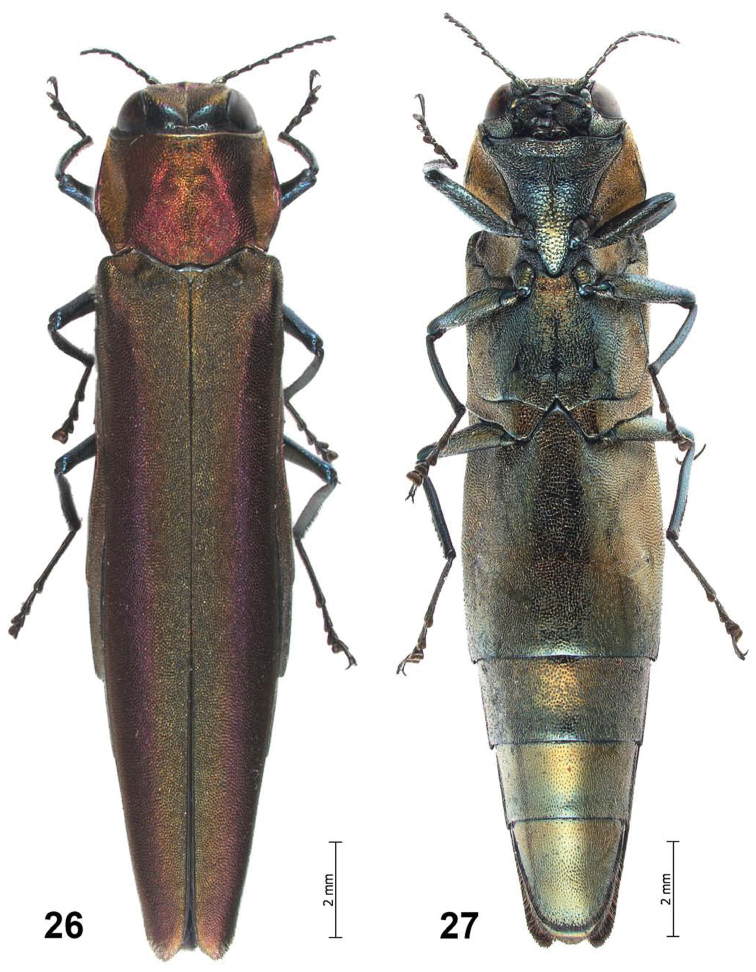
*Agrilus pseudolubopetri* Jendek & Chamorro, sp. n. Female habitus: **26** dorsal view **27** ventral view.

**Figures 28–32. F7:**
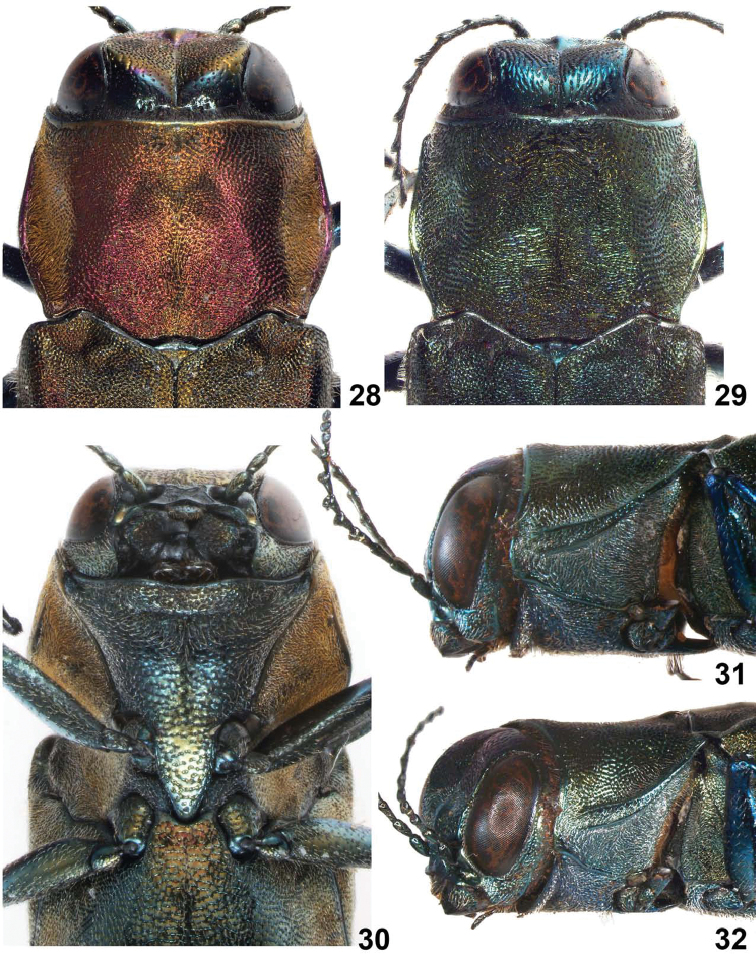
*Agrilus pseudolubopetri* Jendek & Chamorro, sp. n. **28** female, dorsal view of head, pronotum, scutellum **29** male, dorsal view of head, pronotum, scutellum **30** female, ventral view of head and sternum **31** male, lateral view of head and pronotum **32** male, oblique-lateral view of head and pronotum.

**Figures 33–39.**
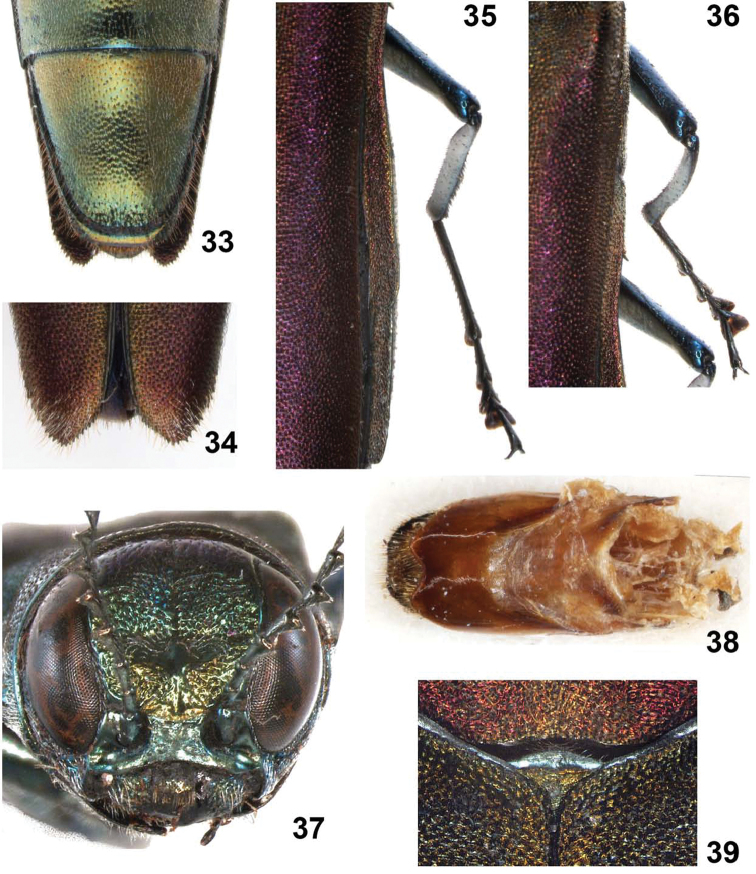
*Agrilus pseudolubopetri* Jendek & Chamorro, sp. n. **33** female, last abdominal ventrite **34** female, apices of elytra **35** female, metathoracic leg **36** female, mesothoracic leg **37** male, anterior view of head **38** female, segment VIII, ventral view **39** female, scutellum.

### Agrilus sapphirinus

Jendek & Chamorro sp. n.

urn:lsid:zoobank.org:act:76C88934-92E3-47B4-BB9D-239FEB7E839B

http://species-id.net/wiki/Agrilus_sapphirinus

Figs 40 50

#### Diagnosis.

This species shares several characters present in species close to *Agrilus ascanius* Deyrolle, 1864. *Agrilus sapphirinus* sp. n. can be easily differentiated by the bright metallic-blue color; lack of yellow abdominal pubescence; strongly convergent marginal and submarginal carinae; and by the dilated prosternal process with acute angles.

**Description**. BODY: Size: 10.5 mm (Holotype); Shape: cuneiform; Build: slender.

HEAD: **Medial impression**: present; Depth: moderately deep; Extent: frons; **Epistoma**: in plane with frons; **Frons**: Shape: flat; Outline: slightly protruding from head outline; **Vertex**: Sculpture **(predominant)**: punctures; Aspect: semispherical; Density: sparse; Intensity: rough; **Eyes**: large; Shape: protruding from head outline; Lower margin: in line with antennal socket; **Antennae**: Length: moderate (females) Shape: slender.

PRONOTUM: Shape: transverse; sides moderately arcuate; widest subapically; anterior margin slightly narrower than posterior; **Anterior lobe** moderate; Shape: arcuate; Position: at level with anterior pronotal angles; **Posterior angles**: Apex: blunt, Shape: moderately obtuse; **Disk**: flat; **Disk impressions**: Presence: medial and lateral; **Medial impression**: Shape: anteromedial and posteromedial; **Lateral impressions**: Depth: deep; Width: wide; **Prehumerus**: Development: carinal; Shape: arcuate; Extent: to third of pronotal length; Anterior end: distant from pronotal angle or margin; Posterior end: distant from pronotal angle or margin; **Marginal and submarginal carinae**: Interspace: narrow; Convergence: strongly convergent; Junction: present; **Scutellum**: Size: moderate, Disk: impressed, Scutellar carina: present.

ELYTRA: Color: unicolored; **Humeral carina**: absent; **Apices**: Arrangement: separate; Shape: spinose; Position of dominant spine: medial; **Elytral pubescence**: absent.

STERNUM: **Prosternal** **lobe**:Size: moderate, Anterior margin: angulately emarginate, Emargination: Depth: deep; Width: wide; **Prosternal process**: Size: moderate; Shape: dilated; Sides: arcuate, Angles: acute, Disk: flat; **Metasternal projection**: flat.

ABDOMEN: **Sternal groove**: Shape on apex of last ventrite: arcuately sinuate; Width: narrow; **Pygidium**: Apical margin: arcuate.

LEGS: Metatarsus: about as long as or somewhat longer than mesotarsus; Metatarsomere1: longer than 2–4 combined.

GENITALIA: **Ovipositor** elongate.

#### Type locality

. North Laos, Louang Namtha environ, N21°00.3, E101°24.6.

#### Type specimens.

**Holotype**, ♀, (EJCB): “LAOS north, 31.v.1997, Luoang Namtha env., N21°00.3, E101°24.6, E. Jendek & O. Šauša leg”.

#### Distribution.

Laos: Louang Namtha Province.

#### Etymology.

The specific epithet is Latin *sapphirinus*, -a, -um (of sapphire) and refers to the color of the species.

**Figures 40–45.**
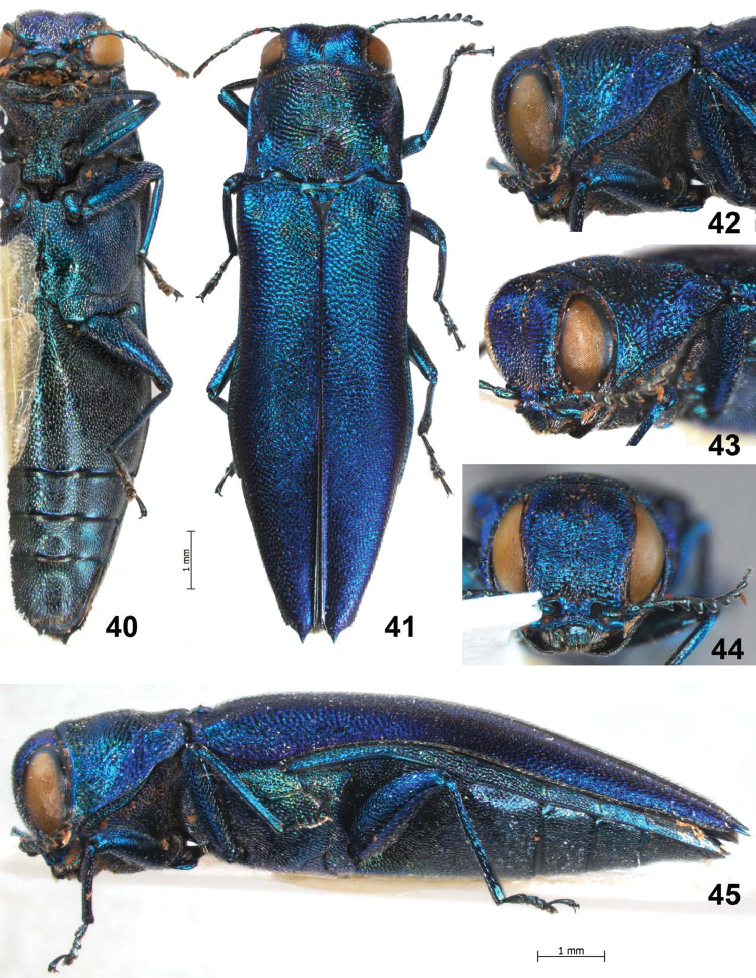
*Agrilus sapphirinus* Jendek & Chamorro, sp. n. Holotype female: **40** ventral view **41** dorsal view **42** lateral view of head and pronotum **43** oblique-lateral view of head and pronotum **44** anterior view of head **45** lateral view.

**Figures 46–50.**
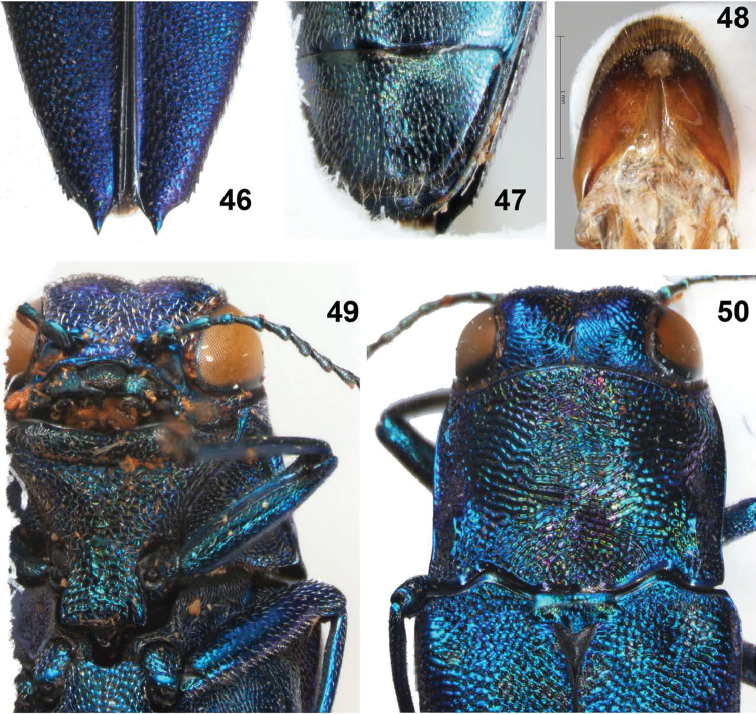
*Agrilus sapphirinus* Jendek & Chamorro, sp. n. Holotype female: **46** elytral apices **47 **last ventrite **48** segment VIII, ventral view **49** ventral view of head and prosternum **50** dorsal view of head, pronotum, and scutellum.

### Agrilus seramensis

Jendek & Chamorro sp. n.

urn:lsid:zoobank.org:act:4086FAB7-63AA-47C7-BA46-2F29F9EACB23

http://species-id.net/wiki/Agrilus_seramensis

Figs 51 60

#### Diagnosis.

This species resembles *Agrilus ascanius* in having the elytra markedly tapering apically; the elytral apices spinose; the pronotum almost square, disk impressions and prehumeral, marginal and submarginal carinae almost identical; a large rectangular scutellum and pronounced triangular scutellar projection; the thorax and abdomen with golden yellow tomentose patterns; and the head obviously large, metallic in color, and eyes markedly protruding. *Agrilus seramensis* can be differentiated by the following characters: the pronotum is green (red in *Agrilus ascanius*), the elytra are green-yellow basally turning blue apically; the scutellum posterior to scutellar carina and scutellar projection depressed; the entire scutellum black; ventrite 2 with lateral tomentose golden-yellow spots; the pronotal lateral margin straight (arcuate in *Agrilus ascanius*); and a broader prosternal process.

#### Description.

BODY: Size: 8.0–11.5 mm (Holotype 11 mm); Shape: cuneiform; Build: slender.

HEAD: Shape: obviously flat; **Medial impression**: Depth: deep; Extent: vertex and frons; **Epistoma**: in plane with frons; **Frons**: Outline: protruding from head outline; **Vertex**:Outline: not protruding from head outline; Sculpture: punctures; Aspect: semispherical, Density: sparse, Intensity: rough; **Eyes**: Size: large; Shape: protruding from head outline; Lower margin: in line with antennal socket; **Antennae**:Length: moderate (females); Shape: slender.

PRONOTUM: Shape: transverse; Sides: straight; **Anterior margin**: narrower than posterior; **Anterior lobe**: moderate; Shape: arcuate; Position: at level with anterior pronotal angles; **Posterior angles**: Apex: sharp, Shape: acute or rectangular; **Disk**: Convexity: flat; **Disk impressions**: Presence: medial and lateral; **Medial impression**: Shape: anteromedial and posteromedial; **Lateral impressions**: Depth: deep; Width: wide; **Prehumerus**: Development: carinal; Extent: to third of pronotal length; **Posterior end**: distant from pronotal angle or margin; **Anterior end**: distant from pronotal marginal carina; **Marginal and submarginal carinae**: Interspace: narrow; Convergence: moderate; Junction: absent; **Scutellum**: Size: moderate, Disk: impressed, Carina: present.

ELYTRA: Color: bicolored; **Humeral carina**: absent; **Apices**: Arrangement: separate; Shape: spinose; Position of dominant spine: medial; **Elytral pubescence**: absent.

STERNUM: **Prosternal** **lobe**: **Size**:moderate; Anterior margin: angulately emarginate; Emargination: depth: deep; Width: wide; **Prosternal process**:Size:moderate; Shape: narrowed; Sides: straight; Angles: obtuse; Disk: flat.

ABDOMEN: **Sternal groove**: Shape on apex of last ventrite: arcuate; **Pygidium**:Apical margin:arcuate.

LEGS: Metatarsus: somewhat longer than mesotarsus; Metatarsomere 1: subequal to or longer than 2–4. combined.

GENITALIA: **Ovipositor**: elongate.

#### Type locality.

Indonesia, Maluku, Seram Island, 35 km East of Pasahari, Unit O.

#### Type specimens.

**Holotype** ♀, (EJCB): “[Indonesia], Maluku, Seram, 35 km E Pasahari, Unit O, 24–30.10.1998, J. Horák leg.” **Paratypes**: 1 ♀, (EJCB): “[Indonesia], Maluku, Seram, Solea, 12 km SE Wahai, 17.i–6.2.1997, S. Bílý leg”. 3 ♀ (EJCB): “[Indonesia], Maluku, Seram, Solea, 12 km SE Wahai, 16.x–4.xi.1998, S. Bílý leg”. 2 ♀ (EJCB) “[Indonesia], Maluku, Seram, Solea, 12 km SE Wahai, 31.10–4.11.1998, J. Horák leg”.

#### Distribution.

Indonesia: Maluku, Seram Island.

#### Etymology.

The specific name is latinized adjective from the geographical term “Seram”, the type locality of this species.

**Figures 51–56.**
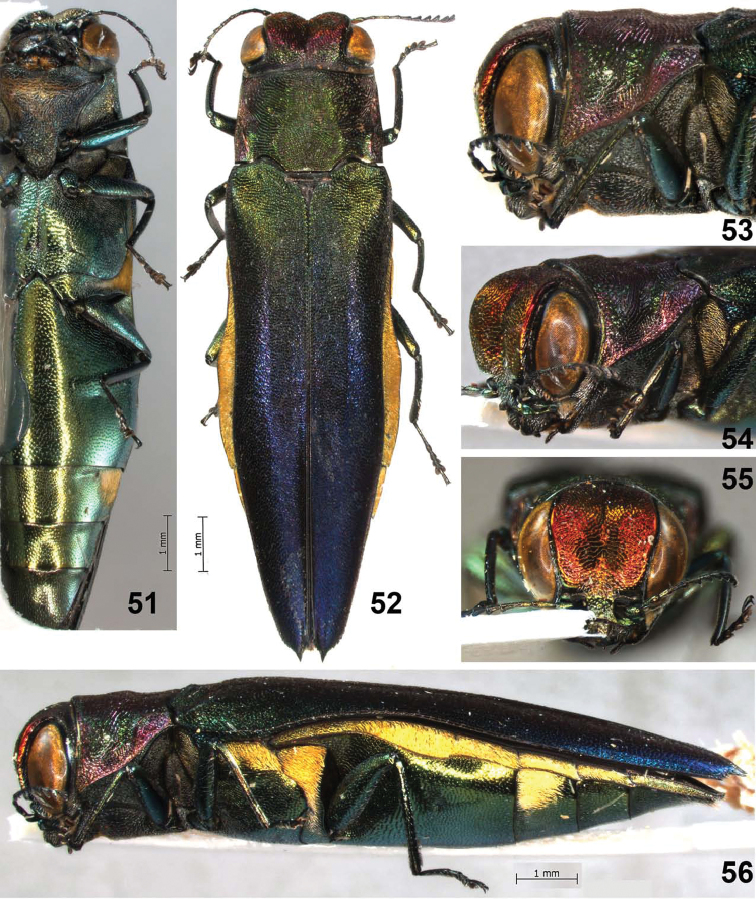
*Agrilus seramensis* Jendek & Chamorro, sp. n. Holotype female: **51** ventral view; **52** dorsal view **53** lateral view head and pronotum **54** oblique-lateral view of head and pronotum **55** anterior view of head **56** lateral view.

**Figures 57–60.**
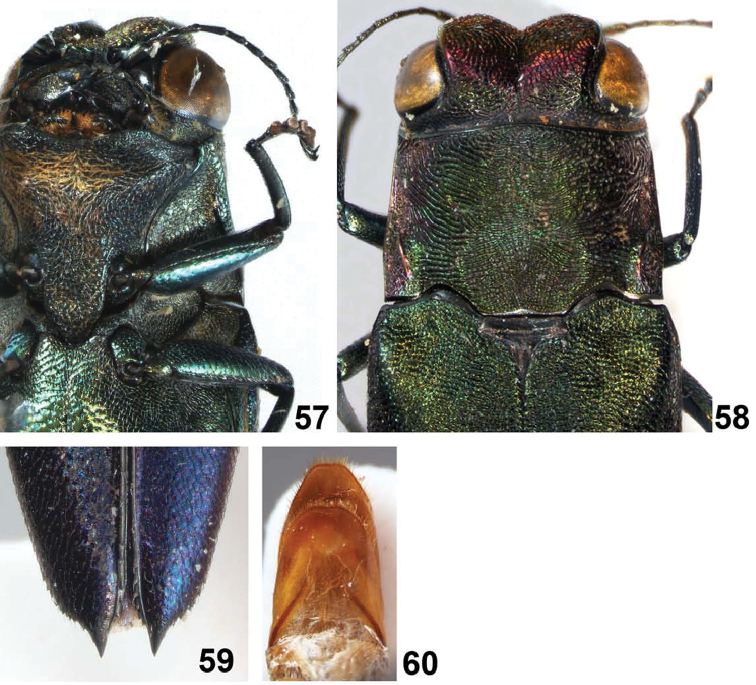
*Agrilus seramensis* Jendek & Chamorro, sp. n. Holotype female: **57** ventral view of head and prosternum **58** dorsal view of head, pronotum, and scutellum **59** elytral apices **60** sternum VIII, ventral view.

**Figures 61–66.**
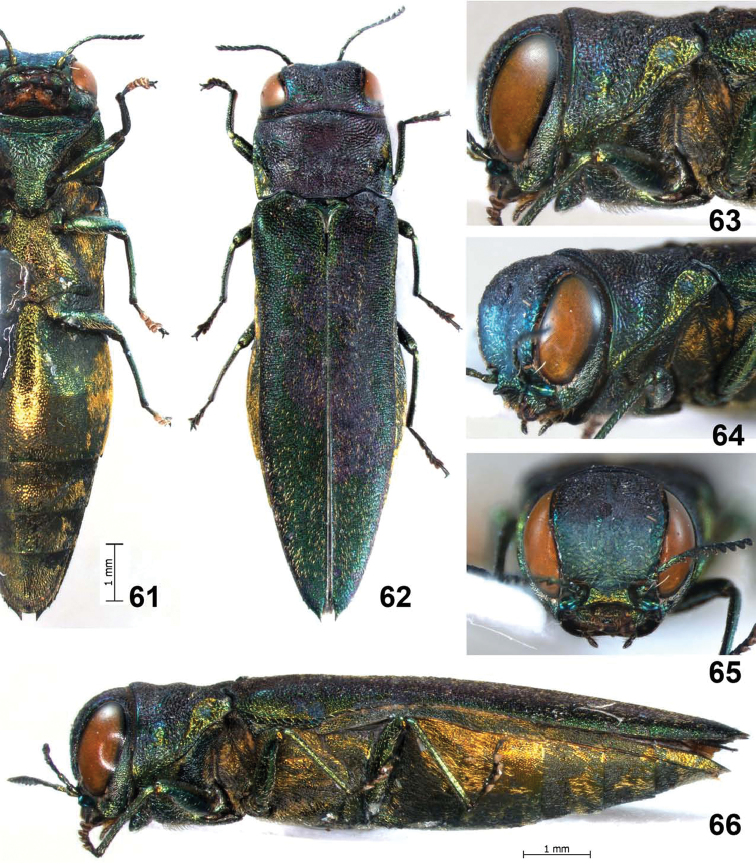
*Agrilus spineus* Jendek & Chamorro, sp. n. Holotype female: **61** ventral view **62** dorsal view **63** lateral view head and pronotum **64** oblique-lateral view of head and pronotum **65** anterior view of head **66** lateral view.

**Figures 67–71.**
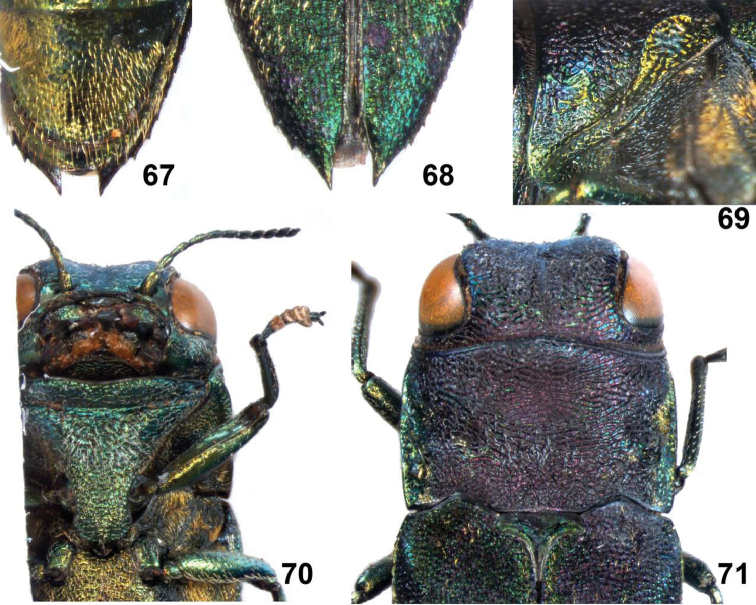
*Agrilus spineus* Jendek & Chamorro, sp. n. Holotype female: **67** last ventrite **68** elytral apices **69** pronotal marginal, submarginal, and prehumeral carinae, oblique-lateral view **70** ventral view of head and prosternum **71** dorsal view of head, pronotum, and scutellum.

### Agrilus spineus

Jendek & Chamorro sp. n.

urn:lsid:zoobank.org:act:95DE2DC1-B948-4FD0-B863-CB527B92B97C

http://species-id.net/wiki/Agrilus_spineus

Figs 61 71

#### Diagnosis.

This species is similar to *Agrilus piliventris* Deyrolle, 1864 in the transverse shape of the pronotum; the ventral and pleural abdominal regions completely covered by golden-yellow pubescence; the scutellum subrectangular with prominent carina; the scutellar disk and carina impressed; the scutellar projection enlarged; and the elytral apices spinose. *Agrilus spineus* can be distinguished from *Agrilus piliventris* and by the metallic black pronotum, greenish-black elytra with minute golden dorsal pubescence; and the elytral apical spines turned medially.

#### Description.

BODY: Size 9 mm (Holotype); Shape: cuneiform; Build: robust.

HEAD: **Medial impression**: present, Extent: frons; **Epistoma**: with raised upper margin; **Frons**: Outline: not protruding from head outline; **Vertex**: Outline: not protruding from head outline; Sculpture: punctures; Aspect: semispherical; Density: sparse; Intensity: rough; **Eyes**: Size: large; Shape: protruding from head outline; Lower margin: below antennal socket; **Antennae**: **Length**: short (female); Shape: slender.

PRONOTUM: Shape: transverse; Sides: arcuate; **Anterior margin**: narrower than posterior; **Anterior lobe**: moderate; Shape: arcuate; Position: at level with anterior pronotal angles; **Posterior angles**: Apex: blunt, Shape: obtuse; **Disk**: flat; **Disk impressions**: Presence: medial and lateral; **Medial** **impression**: Shape: anteromedial and posteromedial; **Lateral** **impressions**: Width: narrow; depth: deep; **Prehumerus**: Development: carinal; Shape: arcuate; Extent: to third of pronotal length; Anterior end: joining with pronotal marginal carina, Posteriorend: distant from pronotal angle or margin; **Marginal and submarginal carinae**: Interspace: narrow; Convergence: strongly convergent; Junction: present; **Scutellum**: Size: moderate; Disk: impressed; Marginal carina: present or obsolete.

ELYTRA: Color: unicolored; Humeral carina: absent; **Apices**: Arrangement: separate; Width: narrow; Shape: spinose; Position of dominant cusp or spine: medial; **Elytral pubescence**: entire.

STERNUM: **Prosternal** **lobe**: **Size**: moderate; Anterior margin: angulately emarginate; Emargination: Depth: deep; Width: wide; **Prosternal process**: Size: moderate; Shape: subparallel; Sides: straight; Angles: obtuse; Disk: flat; **Metasternal projection**: flat.

ABDOMEN: **Sternal groove**: Shape on apex of last ventrite: arcuate; **Pygidium**: Apical margin: arcuate.

LEGS: Metatarsus: about as long as mesotarsus; Metatarsomere 1: subequal to or longer than 2–4 combined.

GENITALIA: **Ovipositor**: elongate.

#### Type locality.

Malaysia, Borneo Island, Sarawak State, Bako National Park.

#### Type specimens.

**Holotype**, ♀, (EJCB): “Borneo, Sarawak, Bako NP, 5.5.2000, M. Vyklický lgt.”.

#### Distribution.

Malaysia: Sarawak state

#### Etymology.

The specific name *spineus* is the Latin adjective spineus, -a, -um (thorny). This refers to the spines on the elytral apices.

### Agrilus tomentipennis

Jendek & Chamorro sp. n.

urn:lsid:zoobank.org:act:775E9A40-700D-4D3B-ABF4-7DF32DF066E0

http://species-id.net/wiki/Agrilus_tomentipennis

Figs 72–80

#### Diagnosis.

*Agrilus tomentipennis* from Laos is very similar to *Agrilus planipennis*. Both species have a small scutellum; identical marginal and submarginal carinae; a pygidial spine; and highly sinuate posterior margin of the metatibiae. While many of the differences between these two species may be considered mostly continuous, such as larger size (*Agrilus tomentipennis*), larger and more pronounced prehumeral carina (*Agrilus tomentipennis*); and deeper frontal concavity (*Agrilus tomentipennis*), two additional features set *Agrilus tomentipennis* apart from *Agrilus planipennis*: the presence of a row of perisutural stripes of white elytral pubescence with indication of a preapical tomentose spot, and a more rectangular scutellum in *Agrilus tomentipennis* (diamond-shaped in *Agrilus planipennis*).

#### Description.

BODY: Size: 14.0–14.3 mm (Holotype 14.3 mm); Shape: cuneiform; Build: robust.

HEAD: Shape: flat; **Medial impression**: present, Depth: deep; Extent: vertex and frons; **Epistoma**: with raised upper margin; **Frons**: Shape: flat; Outline: not protruding from head outline; **Vertex**:Outline: not protruding from head outline; **Sculpture**: Predominant: punctures; Aspect: semispherical, Density: sparse, Intensity: rough; **Eyes**: Size: large, Shape: protruding from head outline; Lower margin: below or in line with antennal socket; **Antennae**: Length: moderate (females); Shape: slender.

PRONOTUM: Shape: visually square to transverse; Sides: arcuate; Maximal width: at middle; Anterior margin: narrower than posterior; **Anterior lobe**: moderate; Shape: arcuate; Position: at level with anterior pronotal angles; **Posterior angles**: Apex: blunt, Shape: obtuse; **Disk**: Convexity: flat, without obvious tomentose spots; **Disk impressions**: Presence: medial and lateral; **Medial impression** (shape): anteromedial and posteromedial; **Lateral impression**: Depth: deep; Width: narrow; **Prehumerus**: Development: carinal; Shape: arcuate; Extent: to third of pronotal length; Anterior end: distant from pronotal angle or margin, Posterior end: joining posterior pronotal margin; **Marginal and submarginal carinae**:Interspace: narrow;Convergence: strong; Junction: present; **Scutellum**: Size: rudimentary, Disk: not impressed, Scutellar carina: obsolete.

ELYTRA: Color: unicolored; Humeral carina: absent; **Apices**: Arrangement: separate; Shape: arcuate; **Elytral pubescence**: perisutural stripes; Color: unicolored; Character: with spots of denser pubescence. STERNUM: **Prosternal** **lobe**: moderate; Anterior margin: arcuately emarginate; Depth: deep; Width: wide. **Prosternal process**: Size: moderatee; Shape: narrowed or subparallel; Sides: straight; Angles: obtuse; **Disk**: flat; **Metasternal** **projection**: flat.

ABDOMEN: **Sternal groove**: Shape on apex of last ventrite: arcuate, **Pygidium**: extended into long spine.

LEGS: **Metatarsus**: distinctly longer than mesotarsus; Metatarsomere 1: subequal to or longer than 2–4 combined.

GENITALIA: Ovipositor: elongate.

#### Type locality.

Northeastern Laos, Xieng Khouang province, 45 km Eastern of Phonsavan: Ban Namseung.

#### Type specimens.

**Holotype** ♀, (EJCB): “LAOS-NE, Xieng Khouang prov., 45 km (by road) E of Phonsavan: ~1000m Ban Namseung, April 2008, Ch. Keomaravong leg”. **Paratypes**: 1 ♀, (USNM): “LAOS-NE, Xieng Khouang prov., 45 km (by road) E. of Phonsavan: ~1000m Ban Namseung, April 2008, Ch. Keomaravong leg”. 1 ♀, (NMPC): “LAOS-NE, Xieng Khouang prov., 45 km (by road) E. of Phonsavan: ~1000m Ban Namseung, vi.2011, Ch. Keomaravong leg”.

#### Distribution.

Laos: Xieng Khouang Province.

#### Etymology.

The specific epithet is a combination of Latin nouns *tomentum* (woolly hairs) and *pennae* (elytra). The name alludes to the presence of white pubescence on the elytra.

#### Remarks.

Jendek and Grebennikov (2011) cited specimens of this taxon in examined material as *Agrilus planipennis*.

**Figures 72–80.**
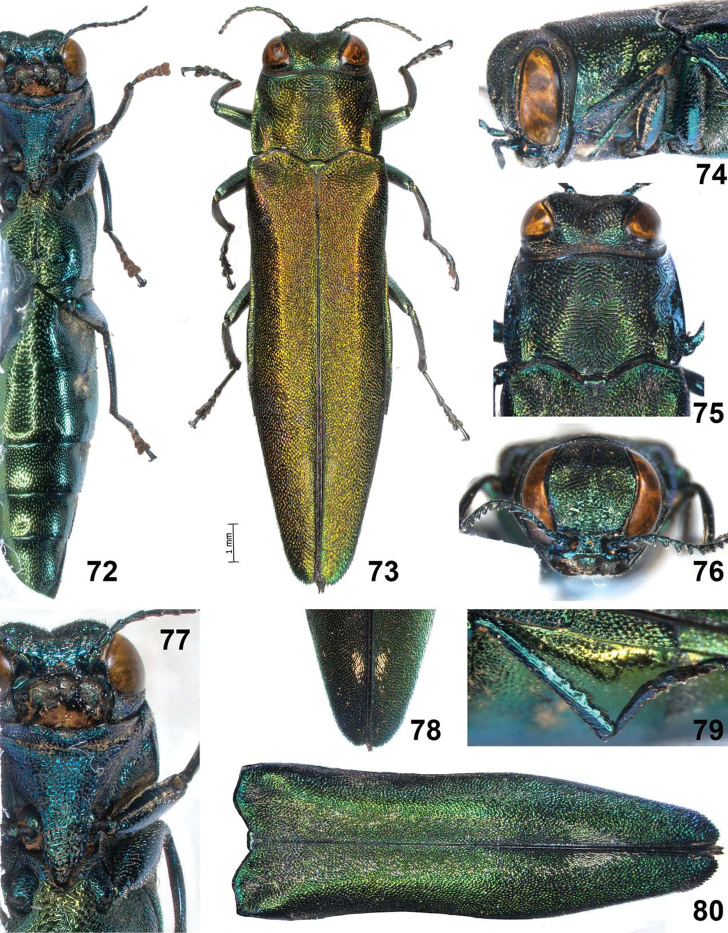
*Agrilus tomentipennis* Jendek & Chamorro, sp. n. Holotype female: **72** ventral view **73** dorsal view **74** lateral view head and pronotum **75** dorsal view of head, pronotum, and scutellum **76** anterior view of head **77** ventral view of head prosternum **78** detail of apex of elytra showing tomentose spots best visible at angle, dorsal view **79** metathoracic leg with sinuate posterior margin **80** elytra, dorsal view (female paratype).

## Supplementary Material

XML Treatment for Agrilus hewitti

XML Treatment for Agrilus daillieri

XML Treatment for Agrilus crepuscularis

XML Treatment for Agrilus pseudolubopetri

XML Treatment for Agrilus sapphirinus

XML Treatment for Agrilus seramensis

XML Treatment for Agrilus spineus

XML Treatment for Agrilus tomentipennis
